# Detection of circRNA Biomarker for Acute Myocardial Infarction Based on System Biological Analysis of RNA Expression

**DOI:** 10.3389/fgene.2021.686116

**Published:** 2021-04-30

**Authors:** Wen Yang, Li Sun, Xun Cao, Luyifei Li, Xin Zhang, Jianqian Li, Hongyan Zhao, Chengchuang Zhan, Yanxiang Zang, Tiankai Li, Li Zhang, Guangzhong Liu, Weimin Li

**Affiliations:** ^1^Department of Cardiology, The First Affiliated Hospital, Harbin Medical University, Harbin, China; ^2^Department of Cardiology, The First Affiliated Hospital, China University of Science and Technology, Hefei, China; ^3^Department of Cardiology, The People’s Hospital of Liaoning Province, Shenyang, China

**Keywords:** circRNA1, AMI 2, microarray 3, bioinformatics 4, circRNA_1047615

## Abstract

Acute myocardial infarction (AMI) is myocardial necrosis caused by the persistent interruption of myocardial blood supply, which has high incidence rate and high mortality in middle-aged and elderly people in the worldwide. Biomarkers play an important role in the early diagnosis and treatment of AMI. Recently, more and more researches confirmed that circRNA may be a potential diagnostic biomarker and therapeutic target for cardiovascular diseases. In this paper, a series of biological analyses were performed to find new effective circRNA biomarkers for AMI. Firstly, the expression levels of circRNAs in blood samples of patients with AMI and those with mild coronary stenosis were compared to reveal circRNAs which were involved in AMI. Then, circRNAs which were significant expressed abnormally in the blood samples of patients with AMI were selected from those circRNAs. Next, a ceRNA network was constructed based on interactions of circRNA, miRNA and mRNA through biological analyses to detect crucial circRNA associated with AMI. Finally, one circRNA was selected as candidate biomarker for AMI. To validate effectivity and efficiency of the candidate biomarker, fluorescence in situ hybridization, hypoxia model of human cardiomyocytes, and knockdown and overexpression analyses were performed on candidate circRNA biomarker. In conclusion, experimental results demonstrated that the candidate circRNA was an effective biomarker for diagnosis and therapy of AMI.

## Introduction

AMI is myocardial necrosis induced by sudden occlusion of a coronary artery (Anderson and Morrow, 2017). In the past few decades, AMI has become a significant cause of emergency medical care, hospitalization, and death in China (Gao et al., 2008; Dai et al., 2017). Globally, the incidence of AMI is increasing year by year with a serious threat to human health and survival quality (Roger et al., 2012). Early diagnosis of AMI is critical for the appropriate initiation of life-saving treatment (Jeong et al., 2020). Biomarkers, such as creatine kinase isoenzyme (CKMB) and troponin I (TnI), are considered the gold standard for AMI. However, early diagnosis of AMI with borderline values of cardiac enzymes or waiting for serial changes could be challenging (Hajar, 2016). Therefore, a better understanding of the pathophysiological mechanisms of AMI and identifying new biomarkers for accurate and specific diagnosis are valuable.

Circular RNA (circRNA) is a type of single-stranded RNA that differs from well-known linear RNA by forming a covalently closed continuous loop (Zeng et al., 2017; Lu et al., 2019). They are generated by back-splicing of pre-mRNA transcripts, in which an upstream splice acceptor is connected to a downstream splice donor (Chen et al., 2019). The closed circular RNA was often considered a by-product of splicing error with little functional potential (Cocquerelle et al., 1993). However, based on the development of high-throughput sequencing, circRNAs have been found abundant, conserved, and specific, implying that they may possess biological and regulatory functions in the cytoplasm (Chen et al., 2019). Currently, circRNAs are found to have the following functions: they can modulate gene expression at the transcriptional or post-transcriptional level by sponging microRNAs (miRNAs) (Hansen et al., 2013; Wei et al., 2014); they can interact with RNA-binding proteins (Du et al., 2016; Wei et al., 2017); and they also have been shown to code for proteins (Begum et al., 2018). circRNA can serve as efficient miRNA sponges, interacting with miRNA to regulate mRNA expression. These specific functions and features of circRNAs suggest that they may be the ideal biomarkers to diagnose some human diseases rapidly.

Circular RNAs have been confirmed to be involved in the development of a variety of diseases (Zeng et al., 2020), including tumor system diseases (Xu et al., 2020), neurological disorders (Rybak-Wolf et al., 2015), endocrine system diseases (Gu et al., 2017), rheumatic system diseases (Luo et al., 2020), and cardiovascular diseases (CVD) (Geng et al., 2016; Wang et al., 2016) observed that circRNA HRCR acted as an endogenous miR-223 sponge to inhibit cardiac hypertrophy and heart failure. (Geng et al., 2016) found that over-expression of circRNA CDR1 *in vivo* increased the cardiac infarct size and suggested the potential of CDR1 was used as a new therapeutic target. These studies implied that circRNA may be a potential diagnostic biomarker and therapeutic target for CVD. However, few studies focused on the effect of circRNA on AMI. This study aimed to investigate the relationship between differentially expressed circRNA and AMI, and reveal the potential mechanisms via circRNA overexpression and knockdown. The ultimate goal was to provide new biomarkers for AMI diagnosis and new target for clinical treatment.

In this study, we performed a series of system biological analysis on RNA expression to find new effective circRNA biomarkers for AMI. The Arraystar Human Circular RNA Microarray Version 2.0 system was employed to detect the differential expression of circular RNAs in the whole blood of 8 patients (4 with acute myocardial infarction (AMI) and 4 with mild coronary artery stenosis). A total of 64 up-regulated and 90 down-regulated circRNAs were identified using traditional statistical methods such as Student two-sample *t* test and fold change. Therefore, five typical down-regulated circRNAs were chosen for RT-qPCR validation. The relative expression levels of 3 circRNAs (068655, 104761, and 104765) were consistent with the results of the microarray. TargetScan and miRanda databases were used to predict interactions between circRNAs and miRNAs. Furthermore, the circRNA-microRNA-mRNA network was constructed. The prediction suggests that the circRNA_104761 can sponge microRNA-449 and microRNA-34a, which are closely correlated with AMI. A larger scale sample experiment observed that the expression of circRNA_104761 was the highest in healthy volunteers, the second highest in mild coronary artery stenosis patients, and the lowest in AMI patients. The area under the receiver operating characteristic (ROC) curve for circRNA_104761 is 0.89, implying a satisfactory prediction accuracy for AMI. To further verify the role of circRNA_104761 in AMI, the hypoxia model of human cardiomyocytes AC16 was established. All the experimental results demonstrated that circRNA_104761 could not only be an effective biomarker for AMI diagnosis, but also differentiate normal coronary artery, mild coronary artery stenosis, and AMI. Furthermore, circRNA_104761 may become a potential therapeutic target.

## Materials and Methods

### Overall Strategy

Abnormally expressed circRNAs often affect the occurrence and development of diseases. To discover the circRNAs related to acute myocardial infarction (AMI), the expression levels of circRNA in blood samples of patients with AMI and those with mild coronary stenosis are firstly analyzed to find abnormally expressed (up-regulated or down-regulated) circRNAs. Next, differentially expressed circRNAs between AMI patients and mild coronary artery stenosis patients were analyzed with hierarchical clustering to find out the similarity of these whole blood samples. Then, RT-qPCR was performed to detect expression levels of circRNAs that were significantly abnormally expressed in blood samples of AMI patients. Afterward, TargetScan and miRanda databases were applied to obtain the data of circRNAs-miRNAs interaction to construct a ceRNA network involving three candidate circRNA biomarkers. Finally, according to the reported data of miRNA regulation of AMI, the circRNA involved in relevant regulation progression was identified and selected circRNA_104761 as candidate biomarker.

**FIGURE 1:**
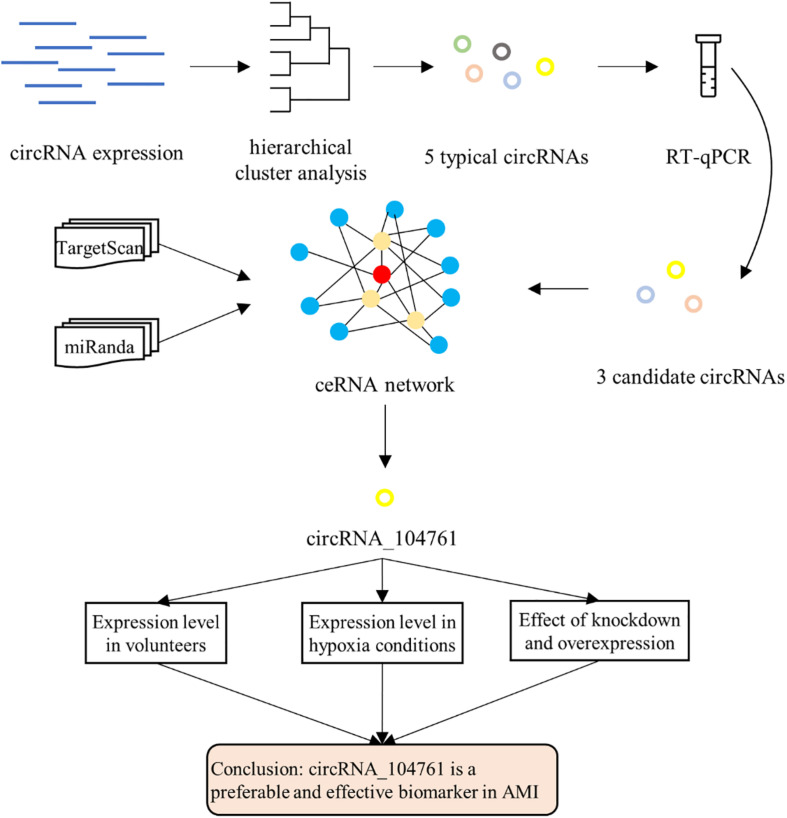
Overall strategy.

To determine the diagnostic potential of the circRNA biomarker selected by above methods for AMI, a series of biochemical experiments were performed. First of all, the expression levels of candidate circRNA biomarker in blood samples of AMI patients, mild coronary artery stenosis patients and normal coronary artery volunteers were detected. The expression levels of candidate circRNA were significantly different in these three groups, and it indicated that the circRNA biomarker was sensitive to AMI and can be used as diagnostic marker. Secondly, hypoxia is a direct consequence of AMI and an important factor leading to death. Subsequently, the expression levels of candidate circRNA biomarker in human cardiomyocytes under different hypoxia conditions were analyzed. The expression levels of candidate circRNA were significantly different under different hypoxia conditions, and it would suggest the circRNA biomarker can be used as molecular marker to determine the pathogenesis of AMI. Finally, the expression levels of candidate circRNA biomarker were intervened by either knockdown or overexpression, identified the influence on the occurrence and development of AMI. The overall strategy was illustrated in Figure 1A.

### Collection of Patient Samples and Ethics Statement

Whole blood samples were collected from 34 AMI patients, 34 patients with mild coronary artery stenosis, and 30 volunteers with normal coronary arteries who attended the First Affiliated Hospital of Harbin Medical University (Harbin, China) in 2019. AMI patients were diagnosed based on acute ischaemic-type chest pain, electrocardiogram (ECG), cardiac enzyme, and coronary angiography, etc. The patients with mild coronary artery stenosis and volunteers with normal coronary arteries were diagnosed by coronary CTA. Patients were excluded from malignant arrhythmia, cardiomyopathy, valvular heart disease, malignant tumors and rheumatic immune system diseases. Blood samples from the AMI patients were collected in 10 min when they arrived at the hospital before taking any medications. Patients with mild coronary artery stenosis and normal coronary artery volunteers were recruited at the time of the fasting blood in the morning. The clinical specimens were obtained from patients who gave informed consent. In the microarray experiment, blood samples from 4 AMI patients and 4 patients with mild coronary artery stenosis were collected. circRNA_104761 was further validated in 30 AMI patients, 30 patients with mild coronary artery stenosis, and 30 volunteers with normal coronary arteries using RT-qPCR. All patients were males, and their age was recorded. The clinical characteristics of the study populations are shown in Supplementary Table 1. The Harbin Medical University ethics committee approved all experimental protocols for the use of human samples, and the methods were carried out in accordance with the approved guidelines.

### Handing and Extraction Total RNA From Human Blood Samples

Whole blood samples (1 mL per patient) were drawn from the study donors via direct venous puncture into 2.0 mL siliconized vacuum tubes containing K2 ethylene diamine tetraacetic acid (EDTA) for Microarray analysis and RT-qPCR. After blood collection, the blood samples were immediately placed into a liquid nitrogen tank and quickly transferred to an ultra-low temperature freezer at −80°C for storage until use. This study extracted total RNA using TRI Reagent BD (Molecular Research Center, OH, United States).

### Microarray Hybridization and Data Analysis

In this study, blood samples from 4 AMI patients and 4 patients with mild coronary artery stenosis were analyzed by the Arraystar Human circRNA Microarray version 2.0 system (Arraystar Inc, Rockville, MD, United States). Total RNA from each sample was quantified using the NanoDrop ND-1000. All samples’ preparation and microarray hybridization were conducted based on the Arraystar’s standard protocols. Briefly, total RNAs were digested with Rnase R (Epicentre, Inc.) to remove linear RNAs and enrich circular RNAs. The enriched circular RNAs were then amplified and transcribed into fluorescent cRNA utilizing a random priming method (Arraystar Super RNA Labeling Kit; Arraystar, MD, United States). The labeled cRNAs were hybridized onto the Arraystar Human circRNA version 2.0 (8x15K, Arraystar). After having washed the slides, the arrays were scanned by the Agilent Scanner G2505C. Agilent Feature Extraction software (version 11.0.1.1) was used to analyze acquired array images. Quantile normalization and subsequent data processing were performed using the R software. Before being used for the cluster analysis, the data were converted to standards. The function of dist and hclust were used to calculate distance and cluster, respectively. Hierarchical Clustering was performed to show the distinguishable expression profile of circRNAs between two groups. Differentially expressed circRNAs with statistical significance between the two groups were identified through Volcano Plot filtering. Differentially expressed circRNAs between two samples were identified through Fold Change filtering.

### RT-qPCR

Total RNA was isolated from 1 mL whole blood using a phenol-chloroform extraction procedure (Jiang et al., 2019; Liu et al., 2019), and RNA extraction process was performed as previously described (Section 2.2). circRNAs’ relative expression level was detected by TB Green Premix Ex Taq II (TaKaRa Bio, Shiga, Japan) with β-actin as an internal control. The validation of all the circRNAs by qPCR was performed ViiA 7 Real-time PCR System (Applied Biosystems). 2^–ΔΔ^ Ct method was used to analyze the RT-qPCR data. Primers used in RT-qPCR for validation are shown in Supplementary Table 2.

### Cell Culture and Hypoxia Models

Human myocardial cell AC16 was purchased from Shenzhen Haodi Huatuo Biotechnology Co Ltd. The basal medium was DMEM medium supplemented with 10% fetal bovine serum (Gibco) and 1% double antibody (100 U/ml penicillin and 100 μg/ml streptomycin, Invitrogen) at 37°C and 5% CO_2_ atmosphere. For the hypoxia experiments, the cells were seeded in the anaerobic mode (oxygen concentration less than 0.1%, carbon dioxide is 5%, and the rest is nitrogen), and then used after 6, 12, and 24 h of treatment.

### Fluorescence *in situ* Hybridization (FISH)

First, AC16 cell climbing slices were fixed in 4% paraformaldehyde (DEPC) for 20 min, shaken, and washed 3 times with PBS (pH 7.4) on a decolorizing shaker, and digested by dropping proteinase K (20 ug/ml) for 8 min. Then, pre-hybridization and hybridization were performed, blocking serum BSA, mouse anti-digoxigenin labeled peroxidase (anti-DIG-HRP), and CY3-TSA was instilled in sequence, and stained with DAPI. Finally, we observed and collected images under fluorescence microscopy.

### Knockdown and Overexpression of circRNA_ 104761

The sequences of siRNA for circRNA_ 104761 used in this study were all synthesized by General Biological System (Anhui) Co Ltd, and three pairs of down-regulation primers were designed for circRNA_ 104761 at the same time. The sequences of primers are shown in Supplementary Table 3. The plasmid vector of circRNA_104761 overexpression model was also synthesized by General Biological System (Anhui) Co Ltd. Cell transfection was performed according to the manufacturer’s instructions.

### CCK-8 Assay, LDH Assay, and Apoptosis Assay

Cell Counting Kits (CCK-8 Kits) were purchased from Tongren Chemical (item number: CK04) to detect cell activity. LDH Assay Kits were purchased from Biyuntian Biotechnology Company (item number: C0017C0017). AnnexinV-FITC/PI Apoptosis Detection Kits were purchased from BD Company (item number: 556547). CCK-8 assay, LDH assay, and Apoptosis assay were all performed according to the kit’s instructions.

### Statistical Analysis

Statistical significance between groups was calculated by Student two-sample *t*-test. The diagnostic value of circRNAs was assessed by receiver operating characteristic (ROC) curves. SPSS statistics version 16.0 software (SPSS Inc, Chicago, IL, United States) was used to do the statistical analysis. A *p*-value < 0.05 was considered to be significant. The Student two-sample *t*-test is expressed as:

(1) $$t\,\left(\upsilon \,\left(i\right)\right)=\frac{m_{2}\,\left(i\right)-m_{1}\,\left(i\right)}{\sqrt{\frac{s_{1}^{2}\,\left(i\right)}{n_{1}}+\frac{s_{2}^{2}\,\left(i\right)}{n_{2}}}},$$

where *i* refers to the ith circRNA. *n*_*1*_ and *n*_*2*_ correspond to sample size of two groups. *m*_1_(*i*) and *m*_2_(*i*) represent the mean values of *i* within the samples in each group. $s_{1}^{2}\,\left(i\right)$ and $s_{2}^{2}\,\left(i\right)$ denotes the corresponding sample variances. υ(*i*) refers to the freedom. That is:

(2) $$\upsilon \,\left(i\right)=\frac{{\left(s_{1}^{2}\,\left(i\right)/n_{1}+s_{2}^{2}\,\left(i\right)/n_{2}\right)}^{2}}{s_{1}^{4}\,\left(i\right)/\left[n_{1}^{2}\cdot \left(n_{1}-1\right)\right]+s_{2}^{4}\,\left(i\right)/\left[n_{2}^{2}\cdot \left(n_{2}-1\right)\right]}.$$

In order to obtain ROC curves and the area under it (Wang et al., 2013; Zhao et al., 2015, 2017; Yang et al., 2020, 2021; Zhai et al., 2020), a certain classifier needs to be assigned. Here, we utilize Fisher’s linear discriminative analysis. A direction vector ***w*** is to be determined, where data ***x*** is to be projected to obtain a value *y*. That is *y* = ***w***^*t*^***x***. The means of the two groups can be expressed as:

(3) $${\text{m}}_{1}=\frac{1}{n_{1}}\,\sum_{x\in D_{1}}\text{x},\,{\text{m}}_{2}=\frac{1}{n_{2}}\,\sum_{x\in D_{2}}\text{x},\,m_{1}=\frac{1}{n_{1}}\,\sum_{x\in D_{1}}{\text{w}}^{t}\,\text{x}={\text{w}}^{t}\,m_{1},\,m_{2}=\frac{1}{n_{2}}\,\sum_{x\in D_{2}}{\text{w}}^{t}\,\text{x}=w^{t}\,{\text{m}}_{2},$$

where *m*_*1*_, *m*_*2*_, *m*_*1*_, and *m*_*2*_ correspond to mean vectors and mean values of the two sample groups before and after projection. Also, we can get:

(4) $$\left|m_{1}-m_{2}\right|=\left|{\text{w}}^{t}\,\left({\text{m}}_{1}-{\text{m}}_{2}\right)\right|.$$

The covariance matrix of samples between classes is:

(5) $${\text{S}}_{B}=\left({\text{m}}_{1}-{\text{m}}_{2}\right)\,{\left({\text{m}}_{1}-{\text{m}}_{2}\right)}^{t}.$$

From equation (4) and equation (5), we can get:

(6) $${\left(m_{1}-m_{2}\right)}^{2}={\left({\text{w}}^{t}\,{\text{m}}_{1}-{\text{w}}^{t}\,{\text{m}}_{2}\right)}^{2}={\text{w}}^{t}\,\left({\text{m}}_{1}-{\text{m}}_{2}\right)\,{\left({\text{m}}_{1}-{\text{m}}_{2}\right)}^{t}\,\text{w}=w^{t}\,{\text{S}}_{B}\,\text{w}.$$

Correspondingly, the covariance matrix of within class samples can be expressed as:

(7) $${\text{S}}_{w}=\sum_{x\in D_{1}}\left(\text{x}-{\text{m}}_{1}\right)\,{\left(\text{x}-{\text{m}}_{1}\right)}^{t}+\sum_{x\in D_{2}}\left(\text{x}-{\text{m}}_{2}\right)\,{\left(\text{x}-{\text{m}}_{2}\right)}^{t}.$$

From equation (7), we can get:

(8) $$\begin{array}{c} s_{1}^{2}+s_{2}^{2}=\sum_{y\in D_{1}}{\left(y-m_{1}\right)}^{2}+\sum_{y\in D_{2}}{\left(y-m_{2}\right)}^{2}\\ \;=\sum_{x\in D_{1}}{\left({\text{w}}_{t}\,{\text{x-w}}_{t}\,{\text{m}}_{1}\right)}^{2}+\sum_{x\in D_{2}}{\left({\text{w}}_{t}\,{\text{x-w}}_{t}\,{\text{m}}_{2}\right)}^{2}\\ \;=\sum_{x\in D_{1}}{\text{w}}^{t}\,\left(\text{x}-{\text{m}}_{1}\right)\,{\left(\text{x}-{\text{m}}_{1}\right)}^{t}\,\text{w}\,+\sum_{x\in D_{2}}{\text{w}}^{t}\,\left(\text{x}-{\text{m}}_{2}\right)\,{\left(\text{x}-{\text{m}}_{2}\right)}^{t}\,\text{w}={\text{w}}^{t}\,{\text{S}}_{w}\,\text{w} \end{array}$$

From equation (6) and equation (8), the Optimization function is expressed as:

(9) $$J\,\left(\text{w}\right)=\frac{{\left(m_{1}-m_{2}\right)}^{2}}{s_{1}^{2}+s_{2}^{2}}=\frac{{\text{w}}^{t}\,{\text{S}}_{B}\,\text{w}}{{\text{w}}^{t}\,{\text{S}}_{w}\,\text{w}}$$

Correspondingly, the best direction for projection can be obtained using following derivation. That is:

(10) $$\begin{array}{c} \frac{\partial J}{\partial \text{w}}=\frac{\left({\text{S}}_{B}+{\text{S}}_{B}^{t}\right)\,\text{w}}{{\text{w}}^{t}\,{\text{S}}_{w}\,\text{w}}-\frac{{\text{w}}^{t}\,{\text{S}}_{B}\,\text{w}\,\left[\left({\text{S}}_{w}+{\text{S}}_{w}^{t}\right)\,\text{w}\right]}{{\left({\text{w}}^{t}\,{\text{S}}_{w}\,\text{w}\right)}^{2}}=0\Leftrightarrow \\ \frac{2\,\left({\text{w}}^{t}\,{\text{S}}_{w}\,\text{w}\right)\,{\text{S}}_{B}\,\text{w}-2\,{\text{w}}^{t}\,{\text{S}}_{B}\,\text{w}\,\left({\text{S}}_{w}\right)\,\text{w}}{{\left({\text{w}}^{t}\,{\text{S}}_{w}\,\text{w}\right)}^{2}}=0\Leftrightarrow \\ {\text{w}}^{t}\,{\text{S}}_{w}\,{\text{wS}}_{B}\,\text{w}={\text{w}}^{t}\,{\text{S}}_{B}\,{\text{wS}}_{w}\,\text{w}\Leftrightarrow \\ \lambda \,{\text{S}}_{w}^{-1}\,{\text{S}}_{B}\,\text{w}=\text{w},\,w\,h\,e\,r\,e\,\,{\text{w}}^{t}\,{\text{S}}_{w}\,\text{w}/{\text{w}}^{t}\,{\text{S}}_{B}\,\text{w}=\lambda \Leftrightarrow \\ \lambda \,{\text{S}}_{w}^{-1}\,\left({\text{m}}_{1}-{\text{m}}_{2}\right)\,{\left({\text{m}}_{1}-{\text{m}}_{2}\right)}^{t}\,\text{w}=\text{w}\Leftrightarrow \\ \text{w}=\lambda^{\prime }\,{\text{S}}_{w}^{-1}\,\left({\text{m}}_{1}-{\text{m}}_{2}\right),\mathrm{where}\,\lambda^{\prime }=\lambda \,{\left({\text{m}}_{1}-{\text{m}}_{2}\right)}^{t}\,\text{w} \end{array}$$

Regarding λ′ as a scalar which can be omitted, the final direction vector *w* can be expressed as:

(11) $$\text{w}={\text{S}}_{w}^{-1}\,\left({\text{m}}_{1}-{\text{m}}_{2}\right).$$

Therefore, Fisher’s linear discriminative analysis can be expressed as:

(12) $${\text{w}}^{t}\,\text{x}+{\text{w}}_{0}=0$$

where ***w***_0_ = ***w***(***m***_1_ + **m**_2_)/2.

## Results

### circRNA Expression Profiles of AMI and Mild Coronary Artery Stenosis Patients

**FIGURE 2:**
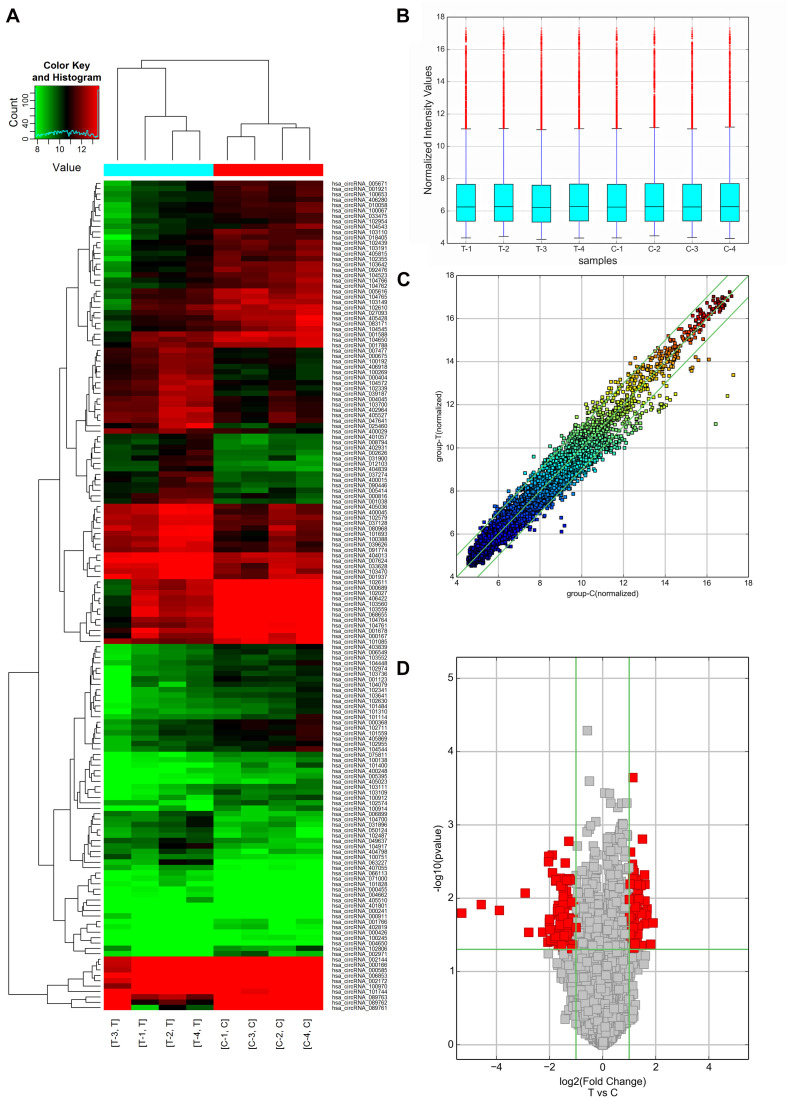
circRNA expression profiles of AMI patients (Group T: T1, T2, T3, T4, and *n* = 4) and mild coronary artery stenosis patients (Group C: C1, C2, C3, C4, and *n* = 4) screened by microarray analysis. **(A)** Heat Map showing a distinguishable expression profile of circRNAs between two groups. Black stands for 0, indicating no change in gene expression; red represents up-regulation, and green represents down-regulation. **(B)** Boxplot view showed the distribution of normalized expression intensity values for two groups. **(C)** Scatter plot indicated the variation of circRNA expression in AMI patients (*y*-axis) and mild coronary artery stenosis patients (*x*-axis). **(D)** Volcano plots visualizing differential circRNA expression between the two groups. The vertical lines correspond to a 2.0-fold change (FC) (log2 scaled) (up-regulation and down-regulation, respectively).

In this study, 64 up-regulated and 90 down-regulated circRNAs were identified in 4 AMI patients compared with 4 mild coronary artery stenosis patients (fold change > 2.0) by microarray analysis (GSE169594), indicating these circRNAs were dysregulation. Differentially expressed circRNAs between AMI patients and mild coronary artery stenosis patients were subjected to hierarchical clustering analysis, suggesting the similarity of these whole blood samples. Hierarchical clustering revealed that the circRNA expression levels were distinguishable in the associated heat map (Figure 2A). A shorter distance generally indicates a high similarity. Therefore, Figure 2A shows that the circRNAs in the AMI patient group, and circRNAs in the mild coronary artery stenosis patient group had a relatively higher similarity. Box plot view (Figure 2B) shows the distribution of the hybridization data and degree of dispersion in AMI patients and mild coronary artery stenosis patients. The box plot shows that after log2 normalization, no abnormal distributions of data were observed in the 8 samples. The scatter and volcano plots shows varied circRNA expressions between the AMI and mild coronary artery stenosis samples (Figures 2C,D). In addition, a volcano plot identified differentially expressed circRNAs at different *p*-values and fold-changes between the two groups.

### *In situ* Validation of the Differentially Expressed circRNAs by RT-qPCR

**FIGURE 3:**
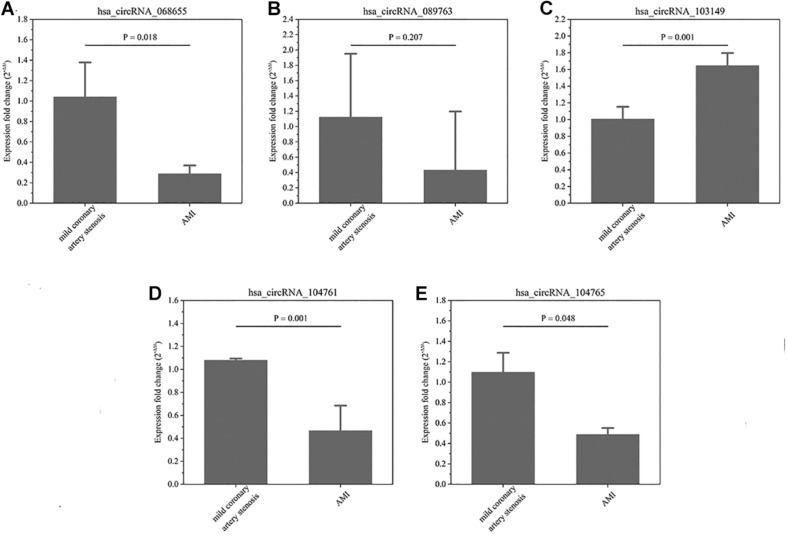
**(A–E)** The relative expression of circRNAs. In-situ verification of the relative expression level of 5 down-regulated circRNAs (068655, 089763, 103149, 104761, and 104765) by RT-qPCR. Results are represented as means ± standard deviation (SD). Data shown in the graphic was analyzed by independent sample *t* tests with a significance level of 95%.

In terms of the microarray results, circRNAs were down-regulated greater than up-regulate, so the down-regulated circRNAs were selected for continued validation. According to circRNA fold change values, P value magnitude, basic intensity of raw signal value (RawIntensity) (recommended above 200), number and sites of circRNA-bound miRNAs and current research status of bound miRNAs, five typical down-regulated circRNAs (hsa_circRNA068655, 089763, 103149, 104761, and 104765) were chosen (shown in Table 1) for further RT-qPCR validation. As shown in Figure 3, the relative expression levels of 4 circRNAs (068655, 089763, 104761, and 104765) were down-regulated in 4 AMI patients, which were consistent with the results of the microarray. However, the *p*-value of circRNA_089763 was over 0.05, which implied a nonsignificant expression difference of circRNA_089763 in two groups. In addition, the relative expression level of circRNA_103149 showed the opposite results of microarray analysis. Therefore, the circRNA_068655, circRNA_104761, and circRNA_104765 could be the potential biomarkers for AMI diagnosis and potential target for AMI treatment.

**TABLE 1:** Five typical down-regulated circRNAs in AMI patients identified by microarray analysis.

circRNAs (has_circRNA)	Alias (has_circRNA)	Fold change	*P*-value	FDR	Regulation	circRNA type	Gene symbol
089763	0089763	14.83	0.014599771	0.684403672	Down	exonic	JA760600
104761	0001847	4.09	0.002673316	0.665381639	Down	exonic	UBAP2
068655	0068655	3.43	0.035551229	0.710909208	Down	exonic	UBXN7
104765	0001850	3.40	0.00561669	0.665381639	Down	exonic	UBAP2
103149	0002903	3.39	0.026855105	0.684403672	Down	exonic	PCNT

### Detailed Annotation for Interaction Between circRNA and miRNA

**FIGURE 4:**
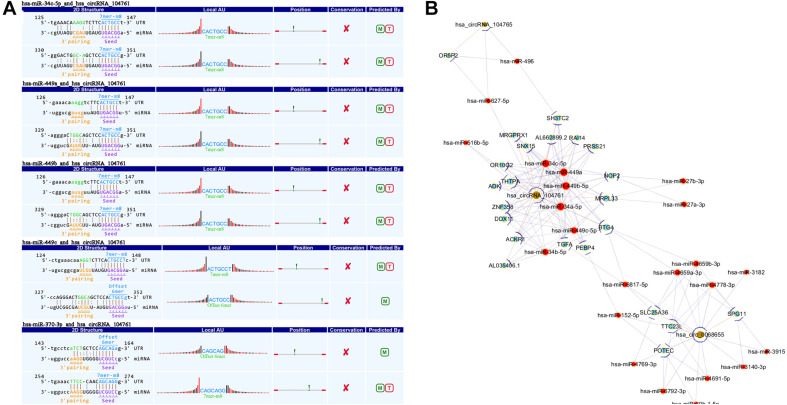
The predicted miRNAs. **(A)** TargetScan and miRanda predicted the 5 most potential target miRNAs that may bind to circRNA _104761 and the binding site’s secondary structure. **(B)** Network of circRNA (068655, 104761, and 104765) -miRNA-mRNA.

This study applied TargetScan and miRanda to find the target miRNA which interacted with circRNAs (068655, 104761, and 104765) and predict the potential biological process in which the discovered circRNAs may participate in. The interaction between circRNAs (068655, 104761, and 104765) and corresponding miRNAs are shown in Table 2. circRNA _104761 may bind potential target miRNAs (hsa-miR-34c-5p, hsa-miRNA-449a, hsa-miRNA 449b-5p, hsa-miRNA-449c-3p, hsa-miR-370-3p) and the secondary structure of the binding site were predicted in Figure 4A. A circRNA-miRNA-mRNA network was built by Cytoscape_3.7.0 as shown in Figure 4B. circRNA_104761 may sponge Hsa-miRNA-34a-5p, Hsa-miR-34b-5p, Hsa-miR-34c-5p, Hsa-miRNA-449a, Hsa-miRNA-449b-5p, and Hsa-miRNA-449c-3p. The circRNA_104761 can sponge microRNA-449 and microRNA-34a which is correlated with AMI (Fan et al., 2013; Zhang et al., 2019). Therefore, circRNA_104761 was selected for further validation in subsequent assays.

**TABLE 2:** TargetScan and miRanda predicted the interaction between circRNA and microRNA.

circRNAs (has_circRNA)	T/C	*p*-value	MRE1	MRE2	MRE3	MRE4	MRE5
068655	0.27	0.00441	hsa-miR-3140-3p	hsa-miR-4539	hsa-miR-3660	hsa-miR-4260	hsa-miR-3118
104761	0.43	0.03983	hsa-miR-34c-5p	hsa-miR-449a	hsa-miR-449b-5p	hsa-miR-449c-5p	hsa-miR-370-3p
104765	0.44	0.04856	hsa-miR-532-5p	hsa-miR-496	hsa-miR-767-5p	hsa-miR-589-5p	hsa-miR-188-3p

### Expression Levels of circRNA_104761 in the Whole Blood of 90 Volunteers

**FIGURE 5:**
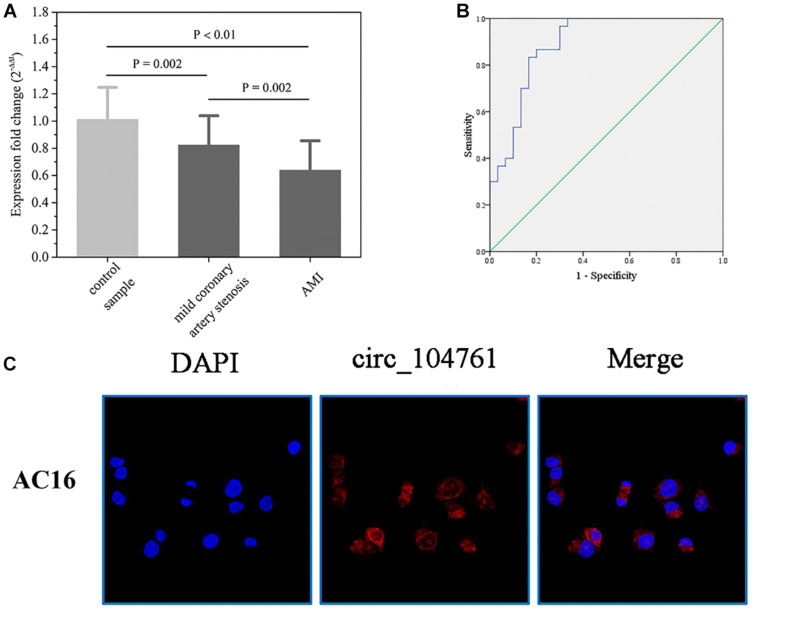
The relative expression of circRNA _104761 in larger scale and the ROC curve analysis. **(A)** Verification of the relative expression level of down-regulated circRNA _104761 by RT-qPCR in expanded sample test. **(B)** ROC curve analysis evaluating the diagnostic value of validated circRNA_104761 for AMI. **(C)** RNA-FISH assays determined the subcellular localization of circRNA_104761 in AC16. Results are represented as means ± standard deviation (SD). Data shown in the graph was analyzed by independent sample *t*-tests with a significance level of 95%.

The differential expressed circRNA_104761 was further validated in 30 AMI patients, 30 mild coronary artery stenosis patients, and 30 normal coronary artery volunteers. As shown in Figure 5A, the average expression level of circRNA_104761 was significantly lower (18%) in AMI patients (0.639 ± 0.217)) than mild coronary artery stenosis patients (0.824 ± 0.216, *p* = 0.002), suggesting that the predicated circRNA by microarray was effective in a larger scale sample. Besides, it is worthy to note that the expression difference of circRNA_104761 between mild coronary artery stenosis group (0.824 ± 0.216) and normal coronary artery group (1.012 ± 0.235) was also significant (*p* = 0.002), which implied the expression of circRNA_104761 in mild coronary artery stenosis patients had been inhibited. The expression of circRNA_104761 was the highest in normal coronary artery volunteers, the second highest in mild coronary artery stenosis patients, and the lowest in AMI patients. The median cycle threshold (Ct) value for circRNA_104761 in 90 samples was 29.027, ranging from 27.623 to 32.971. These results suggest that circRNA_104761 is sensitive and abundant in human blood.

### ROC Analysis of Validated circRNAs in AMI Patients and FISH

The receiver operating characteristic (ROC) curves and the area under the ROC curve (AUC) were used to confirm the relationship between circRNA_104761 and AMI. As shown in Figure 5B, the AUC value of the ROC curve for circRNA_104761 was 0.890 (95% confidence interval [CI] = 0.807–0.973). Meanwhile, the sensitivity and specificity of the circRNA_104761 ROC curve were 0.867 and 0.800, respectively. These results indicated that circRNA_104761 can be considered a preferable and effective biomarker for the diagnosis of AMI. RNA-FISH assay reveals that circRNA_104761 (FAM-labeled) distinctly distributed in the cytoplasm of AC16 cells (Figure 5C).

### Expression of circRNA_104761 in Hypoxic Human Cardiomyocytes AC16 and the circRNA_104761 Editing Efficiency

**FIGURE 6:**
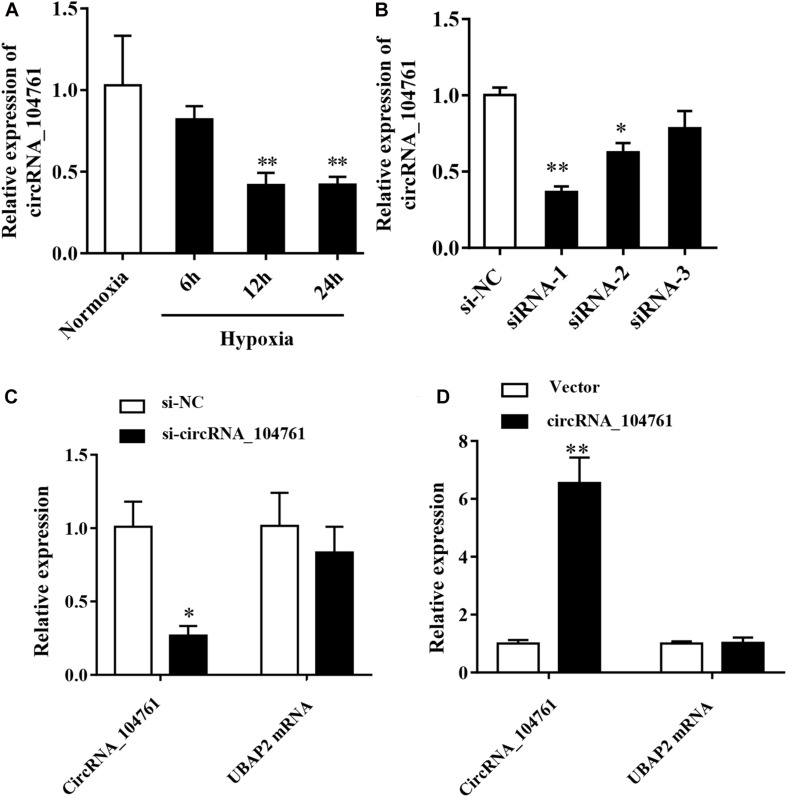
Expression of circRNA_104761 in hypoxic human cardiomyocytes AC16 and the circRNA_104761 editing efficiency. **(A)** Expression of circRNA _ 104761 in AC16. **(B)** The down-regulation efficiency of three siRNAs (siRNA-1, siRNA-2, and siRNA-3). **(C)** The effect of siRNA-1 on endogenous circRNA_104761 and its source gene UBAP2. **(D)** The over-expression efficiencies of circRNA_104761 and UBAP2. *p < 0.05 and **p < 0.01.

Hypoxia is an important factor causing myocardial injury. After cardiomyocytes AC16 were treated with hypoxia condition for 6 h (6 h group), 12 h (12 h group), and 24 h (24 h group), and the expression of circRNA_104761 was detected by RT-qPCR. The results showed that the expression level of circRNA_104761 in hypoxic cardiomyocytes AC16 (Hypoxia, 6, 12, and 24h) was inhibited compared with normal AC16 (Normoxia), and the expression level of circRNA_104761 in hypoxic cardiomyocytes AC16 (12h, 24h) was significantly decreased (Figure 6A, *p* < 0.01). Hypoxia treatment for 12 h was chosen for subsequent experiments. To further investigate the effect of circRNA_104761 on cardiomyocytes, we constructed siRNAs that could knockdown the expression of circRNA_104761. This study designed three pairs of siRNAs (siRNA-1, siRNA-2, and siRNA-3) to verify the down-regulation efficiency by RT-qPCR. The results showed that siRNA-1 and siRNA-2 down-regulation efficiency was significant (Figure 6B). Moreover, siRNA-1 was selected for further experiments. The source gene of circRNA_104761 was UBAP2 mRNA by circbase query, and specific siRNA-1 was able to significantly interfere with endogenous circRNA_104761, but had no significant effect on its source gene (UBAP2) (Figure 6C). Furthermore, we constructed a plasmid vector to overexpress circRNA_104761 in AC 16, and RT-qPCR was used to verify the overexpression efficiency. The results showed that the overexpression efficiency of the constructed plasmid vector was significant. The specific plasmid vector could significantly overexpress circRNA_104761, but had no significant effect on its source gene (UBAP2) (Figure 6D). Therefore, this plasmid vector was used for the following experiments.

### Effect of circRNA_104761 Knockdown and Overpression in Human Cardiomyocytes AC16

**FIGURE 7:**
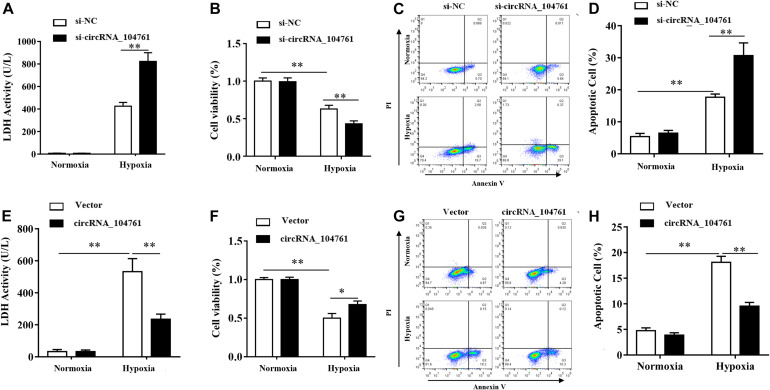
Effect of circRNA_104761 knockdown and overpression in human cardiomyocytes AC16. **(A–D)** Effect of circRNA_104761 knockdown on AC16. **(A)** LDH activity test (Normoxia and Hypoxia). **(B)** Cell viability was determined by CCK8 assay. **(C,D)** Flow data analysis of apoptosis. **(E–H)** Effect of circRNA_104761 overexpression on AC16. **(E)** LDH activity test (Normoxia and Hypoxia). **(F)** Cell viability was determined by CCK8 assay. **(G,H)** Flow data analysis of apoptosis. Results are represented as means ± standard deviation (SD). Data shown in this graph was analyzed by independent sample *t*-tests with a significance level of 95%. **p-value < 0.01 and *p-value < 0.05.

After knockdown of circRNA_104761 with siRNA, LDH assay demonstrated LDH activity increased after hypoxia treatment, and that down-regulation of circRNA_104761 significantly increased the release of lactate dehydrogenase in AC16 cell after hypoxia treatment (Figure 7A, *p*-value < 0.01). CCK8 assay demonstrated cell viability was significantly lower after hypoxia treatment, and that down-regulation of circRNA_104761 significantly reduced AC16 cell viability after hypoxia condition (Figure 7B), which enhanced AC16 cell death. Flow cytometry data demonstrated apoptosis was significantly increased after hypoxia, and that down-regulation of circRNA_104761 significantly exacerbated the early apoptotic level of AC16 cells after hypoxia treatment (Figures 7C,D).

When the expression of circRNA_104761 was overexpressed by the constructed plasmid vector, LDH activity was significantly increased after hypoxia, and the results of LDH tests demonstrated that exogenous overexpression of circRNA_104761 significantly decreased the release of lactate dehydrogenase in AC16 cell after hypoxia treatment (Figure 7E, *p* < 0.01). CCK8 assay demonstrated cell viability was significantly lower after hypoxia treatment, and that the overexpression of circRNA_104761 significantly increased AC16 cell viability after hypoxia treatment (Figure 7F). Flow cytometry data demonstrated apoptosis significantly increased after hypoxia treatment, and that overexpression of circRNA_104761 significantly alleviated the early apoptotic level of AC16 cell after hypoxia treatment (Figures 7G,H).

## Discussion

For the first time, our study applied microarray to identify the differences in circRNA expression levels between AMI patients and mild coronary artery stenosis patients. Results of microarray analysis were validated by RT-qPCR in larger samples (30 AMI patients, 30 mild coronary artery stenosis patients, and 30 normal coronary artery volunteers) and in human cardiomyocytes AC16, which implied that the expression of circRNA_104761 was an effective biomarker for AMI diagnosis. Given that circRNAs and miRNAs interact each other, circRNAs may be involved in the biological process of AMI through sponge miRNAs (Tang et al., 2018; Faiza et al., 2019; Chowdhury et al., 2020; Khan et al., 2020). The target microRNAs of circRNA_104761 were predicted by TargetScan, miRanda, and circRNA–microRNA-mRNA network, and we found that circRNA_104761 could sponge microRNA-449 and microRNA-34a. It is worth noting that miRNA-449 and miRNA-34a are closely linked to AMI.

circRNA_ 104761 may promote cardiomyocyte apoptosis through sponging miR-449. In the study of Zhang et al. (2019), they found that knocking down lncRNAX inactivation specific transcript (XIST) in the AMI rat model could down-regulate the level of miRNA-449 and inhibit rat cardiomyocyte apoptosis, suggesting that miRNA-449 is directly involved in the regulation of MI. MiRNA-449 regulate gene expression post-transcriptionally through mRNA degradation or translational repression (Esquela-Kerscher and Slack, 2006). MiRNA-449 is down-regulated in various cancers and is a strong inducer of cell cycle arrest (including senescence) and apoptosis in tumor cell lines (Bou Kheir et al., 2011). MiRNA-449 regulates various pathways (Lize et al., 2011), including Notch (Marcet et al., 2011), p53, E2F1 (Lize et al., 2010; Noonan et al., 2010),Wnt (Iliopoulos et al., 2009), and cell cycle (Bou Kheir et al., 2011). Among them, miRNA-449 provides negative feedback on E2F pathway and positive feedback on the p53 pathway, strengthening E2F1-p53 interdependence (Lize et al., 2010). In response to DNA damage, the transcription p53 and E2F1 deregulated in cancer, and then they were activated to induce pro-apoptotic genes, which directly promote apoptosis (Lize et al., 2011). circRNA_104761 is down-regulated in AMI, which may reduce sponge miRNA-449 and change p53 and E2F-1 pathways, increasing myocardial apoptosis.

Furthermore, circRNA_ 104761 may promote cardiomyocyte apoptosis through sponging miR-34a. Fan et al. (2013) observed that miRNA-34a promoted cardiomyocyte apoptosis by negatively regulating aldehyde dehydrogenase 2 (ALDH2), which was increased in circulation under myocardial infarction (MI) conditions. In addition, the miR-34a can act as p53-responsive genes, which can induce apoptosis and cell cycle arrest in tumor cell lines (Rockenfeller et al., 2010). MiRNA-34a regulates many target proteins, which induce cell apoptosis in p53-dependent manner, including bcl-2 (Cole et al., 2008), YY1 (Chen et al., 2011), Notch (Li et al., 2009), MAPK (Tivnan et al., 2011), and DLL1 (Lewis et al., 2003), or independent manner. In AMI, the expression of circRNA_104761 is down-regulated, which may reduce the sponge function of circRNA_104761 on miRNA-34a, resulting in changes in the p53-miRNA-34a axis, causing myocardial apoptosis.

There are several limitations in our study, which cannot be ignored. First, the number of subjects is not large enough, limiting the clinical value of circRNA_104761 as a potential biomarker. Also, a more diverse control group is needed, such as patients with moderate coronary artery stenosis and patients with severe coronary artery stenosis. Second, to further illustrate the application value of circRNA_104761 in AMI, animal models with knockdown or overexpression of circRNA_104761 are needed, and this experiment only carried out cell verification. In addition, due to limited funding, we only speculated the mechanism of circRNA_104761 via miRNA-449 and miRNA-34a to cause AMI by functional analysis. The relationship between circRNA_104761 and miRNA-499/miRNA-34a needs further investigation and verification. Finally, restricting the population to Asian males limited the generalizability of the findings to females and other races.

In summary, our results demonstrated that circRNA_104761 could not only be an effective biomarker for AMI diagnosis, but also differentiate normal coronary artery, mild coronary artery stenosis, and AMI. This study also identified that knockdown of circRNA_104761 with siRNA aggravated hypoxia-induced cardiomyocytes injury in AC16, and overexpression of circRNA_104761 alleviated hypoxia-induced injury. Therefore, circRNA_104761 may be considered a potential therapeutic target.

## Data Availability Statement

The original contributions presented in the study are included in the article/Supplementary Material, further inquiries can be directed to the corresponding author.

## Ethics Statement

The studies involving human participants were reviewed and approved by the Harbin Medical University Ethics Committee. The patients/participants provided their written informed consent to participate in this study.

## Author Contributions

WY, LS, XC, LL, XZ, and WL conceived, designed the study, and revised the manuscript. WY, JL, TL, LZ, and GL collected samples. WY, HZ, CZ, and YZ performed the experiments and analyzed the data. All authors approved the final version of the manuscript.

## Conflict of Interest

The authors declare that the research was conducted in the absence of any commercial or financial relationships that could be construed as a potential conflict of interest.
