# Supplementary material for: Detection of circRNA Biomarker for Acute Myocardial Infarction Based on System Biological Analysis of RNA Expression
Source: Front Genet. 2021 Apr 30;12:686116. doi: 10.3389/fgene.2021.686116 (PMC8120315; doi:10.3389/fgene.2021.686116)
Supplement: Supplementary file 1 [file Data_Sheet_1.docx]

Supplementary Material

# Supplementary Tables

**Table S1. Clinical characteristics of patients.**

|  | **Microarray analysis** | | | **RT-qPCR validation** | | | | | |  |
| --- | --- | --- | --- | --- | --- | --- | --- | --- | --- | --- |
| **Characteristics** | AMI (n=4) | Mild coronary artery stenosis (n=4) | *P* | AMI (n=30) | Mild coronary artery stenosis (n=30) | Normal coronary artery (n=30) | *P_1_* | *P_2_* | *P_3_* | |
| **Age (years)** |  |  |  |  |  |  | 0.923 | 0.004 | 0.002 | |
| Total (missing) | 4 (0) | 4 (0) | 0.54 | 30 (0) | 30 (0) | 30 (0) |  |  |  |  |
| Mean (SD) | 52  (10.17 ) | 48  (6.98) |  | 59.87  (14.37 ) | 59.53  (12.08 ) | 49.93  (10.59) |  |  |  |  |
| Median (IQR) | 55  (55-59.3 ) | 47.5  (42-53.5) |  | 58  (48.5-70.8) | 62  (52.25-67.8) | 54  (42-57.0) |  |  |  |  |
| **Male/female (n/n)** | (4/0) | (4/0) |  | (30/0) | (30/0) | (30/0) |  |  |  | |
| **Hypertension History** | |  |  |  |  |  |  |  |  | |
| Yes | 0 | 1 |  | 15 | 8 | 2 |  |  |  |  |
| No | 4 | 3 |  | 15 | 22 | 28 |  |  |  |  |
| Total (missing) | 4 (0) | 4 (0) |  | 30 (0) | 30 (0) | 30 (0) |  |  |  |  |
| **Smoking History** |  |  |  |  |  |  |  |  |  | |
| Yes | 4 | 4 |  | 19 | 16 | 0 |  |  |  |  |
| No | 0 | 0 |  | 11 | 14 | 30 |  |  |  |  |
| Total (missing) | 4 (0) | 4 (0) |  | 30 (0) | 30 (0) | 30 (0) |  |  |  |  |
| **Drinking** **history** |  |  |  |  |  |  |  |  |  | |
| Yes | 1 | 3 |  | 10 | 13 | 0 |  |  |  |  |
| No | 3 | 1 |  | 20 | 17 | 30 |  |  |  |  |
| Total (missing) | 4 (0) | 4 (0) |  | 30 (0) | 30 (0) | 30 (0) |  |  |  |  |
| **Fasting glucose (mmol/L)** | |  |  |  |  |  | 0.186 | 0.471 | 0.042 | |
| Mean (SD) | 7.82  (2.86) | 8.68  (6.97) | 0.829 | 6.11  (1.65) | 6.76  (2.06) | 5.83  (1.23) |  |  |  |  |
| Median (IQR) | 6.625  (6.293-8.2 ) | 5.435  (4.815-9.3) |  | 5.79  (5.79-6.7) | 6.09  (5.24-7.7) | 5.6  (5.09-6.4 ) |  |  |  |  |
| Total (missing) | 4(0) | 4(0) |  | 29 (1) | 29 (1) | 29 (1) |  |  |  |  |
| **SBP (mmHg)** |  |  |  |  |  |  | 0.127 | 0.807 | 0.077 | |
| Mean (SD) | 146.25(14.24) | 127.50  (17.08) | 0.143 | 130.33  (29.19) | 140.79  (19.87) | 131.93  (17.58) |  |  |  |  |
| Median (IQR) | 147(135-158.3) | 125  (117.5-135.0) |  | 124  (109-151.5) | 140  (130-158) | 130  (121-143) |  |  |  |  |
| Total (missing) | 4 (0) | 4 (0) |  | 27 (3) | 29 (1) | 29 (1) |  |  |  |  |
| **DBP (mmHg)** |  |  |  |  |  |  | 0.619 | 0.659 | 0.904 | |
| Mean (SD) | 101.75  (12.15) | 87.50  (9.57) | 0.115 | 84.3  (18.79) | 86.55  (14.84) | 86.14  (10.9) |  |  |  |  |
| Median (IQR) | 101  (92.3-110.5) | 85  (80.0-92.5) |  | 83  (72.5-95.5) | 90  (70-100) | 84  (80-92) |  |  |  |  |
| Total (missing) | 4 (0) | 4 (0) |  | 27 (3) | 29 (1) | 29 (1) |  |  |  |  |
| **TC (mmol/L)** |  |  |  |  |  |  | 0.559 | 0.518 | 0.254 | |
| Mean (SD) | 6.30  (0.73) | 5.80  (1.15) | 0.492 | 4.5  (0.89) | 4.35  (1.02) | 4.679  (0.96） |  |  |  |  |
| Median (IQR) | 6.4  (6.048 -6.650) | 6.175  (5.563-6.413) |  | 4.61  (4.115-5.0) | 4.29  (3.75-4.7) | 4.67  (3.89-5.2) |  |  |  |  |
| Total (missing) | 4 (0) | 4 (0) |  | 28 (2) | 30 (0) | 23 (7) |  |  |  |  |
| **TG (mmol/L)** |  |  |  |  |  |  | 0.577 | 0.916 | 0.494 | |
| Mean (SD) | 2.45  (1.33) | 3.16  (1.12) | 0.442 | 2.42  (2.03) | 2.16  (1.48) | 2.48  (1.9) |  |  |  |  |
| Median (IQR) | 2.66  (2.1025-3.0) | 3.38  (2.48-4.1) |  | 1.81  (1.1-3.1) | 1.815  (1.17-2.5) | 1.98  (1.5-2.5) |  |  |  |  |
| Total (missing) | 4 (0) | 4 (0) |  | 28 (2) | 30 (0) | 23 (7) |  |  |  |  |
| **HDL (mmol/L)** |  |  |  |  |  |  | 0.345 | 0.985 | 0.333 | |
| Mean (SD) | 1.08  (0.22) | 1.25  (0.35 ) | 0.443 | 1.17  (0.44) | 1.08  (0.23) | 1.16  (0.39) |  |  |  |  |
| Median (IQR) | 1.045  (0.935-1.2) | 1.235  (1.123 -1.4) |  | 1.05  (0.975-1.3) | 1.055  (0.923-1.2) | 1.12  (0.935-1.2) |  |  |  |  |
| Total (missing) | 4 (0) | 4 (0) |  | 28 (2) | 30 (0) | 23 (7) |  |  |  |  |
| **LDL (mmol/L)** |  |  |  |  |  |  | 0.944 | 0.844 | 0.790 | |
| Mean (SD) | 4.04  (0.53） | 2.58  (1.72) | 0.189 | 2.8  (0.63) | 2.82  (0.62) | 2.77  (0.67) |  |  |  |  |
| Median (IQR) | 4.16  (3.935-4.3) | 2.565  (1.235-3.9) |  | 2.83  (2.54-3.1) | 2.78  (2.43-3.0) | 2.74  (2.315-3.4) |  |  |  |  |
| Total (missing) | 4 (0) | 4 (0) |  | 28 (2) | 30 (0) | 23 (7) |  |  |  |  |
| **AST (U/L)** |  |  |  |  |  |  | 0.01 | 0.003 | 0.432 | |
| Mean (SD) | 103.03  (79.36) | 28.80  (19.50) | 0.157 | 59.16  (61.3) | 26.28  (16.01 ) | 23.33  (12.43) |  |  |  |  |
| Median (IQR) | 87.05  (39.25-150.8) | 20.9  (17.95-31.8) |  | 36  (25.25-64.5) | 23.5  (16.7-32.1) | 18.35  (16.45-24.8) |  |  |  |  |
| Total (missing) | 4 (0) | 4 (0) |  | 28 (2) | 29 (1) | 30 (0) |  |  |  |  |
| **ALT (U/L)** |  |  |  |  |  |  | 0.071 | 0.012 | 0.415 | |
| Mean (SD) | 38.53  (19.41) | 25.55  (4.02) | 0.238 | 43.13  (26.1) | 31.50  (21.34) | 26.95  (21.21) |  |  |  |  |
| Median (IQR) | 36.5  (30.5-44.5) | 25.05  (22.35-28.3) |  | 37.5  (28.75-48.3) | 24  (17.6-39.4) | 20.3  (14.35-30.7) |  |  |  |  |
| Total (missing) | 4 (0) | 4 (0) |  | 28 (2) | 29 (1) | 30 (0) |  |  |  |  |
| **SCR (umol/l)** |  |  |  |  |  |  | 0.128 | 0.042 | 0.385 | |
| Mean (SD) | 88.10  (14.57) | 71.55  (16.79) | 0.187 | 88.44  （36.36 ） | 76.88  (17.40) | 73.70  (8.73) |  |  |  |  |
| Median (IQR) | 83.55  (77.475-94.2) | 67.2  (60.8-78.0) |  | 82.7  (73.8-88.9) | 75.7  (61.8-86.7) | 71.65  (67.4-78.5) |  |  |  |  |
| Total (missing) | 4 (0) | 4 (0) |  | 29 (1) | 29 (1) | 28 (2) |  |  |  |  |
| **UA (umol/l)** |  |  |  |  |  |  | 0.359 | 0.615 | 0.073 | |
| Mean (SD) | 407.30  (40.63) | 363.13  (99.97) | 0.444 | 381.93  (101.21) | 359.46  (83.00) | 392.58  (49.41) |  |  |  |  |
| Median (IQR) | 422.8  (398.7-431.4) | 340.85  (321.1-382.9) |  | 362.3  (327.5-401.3) | 349.1  (310.4-434.0) | 388.95  (359.75-419.8) |  |  |  |  |
| Total (missing) | 4 (0) | 4 (0) |  | 29 (1) | 29 (1) | 28 (2) |  |  |  |  |

SBP, systolic blood pressure; DBP, diastolic blood pressure; TC, total cholesterol; TG, total glyceride; HDL, high-density lipoprotein; LDL, low-density lipoprotein; AST, Aspartate aminotransferase; ALT, Alanine aminotransferase; SCR, Serum creatinine; UA, Uricacid. *P*: comparison between patients with AMI and mild coronary artery stenosis in microarray analysis. *P_1_*: comparison between patients with AMI and mild coronary artery stenosis in RT-qPCR validation. *P_2_*: comparison between patients with AMI and normal coronary artery in RT-qPCR validation. *P_3_*: comparison between patients with mild coronary artery stenosis and normal coronary artery in RT-qPCR validation.

**Table S2. Primers used in RT-qPCR for validation.**

| *Name (has*_*circRNA)* | *Primer sequences* | *Product size (bp)* |
| --- | --- | --- |
| 068655 | F:5’ TCCTCAAAAGCAGGAAATACTG 3’  R:5’ CTACACTTTCACTTGCACCACC 3’ | 65 |
| 089763 | F:5’ CAATGGTGAGGGAGGTAGGT 3’  R:5’ ACTCCTAATCACATAAATGCCC 3’ | 136 |
| 103149 | F:5’ GTCATCATCAAGCAGCCATT 3’  R:5’ AGATTCCCAATCTTCCTTCAG 3’ | 140 |
| 104761 | F:5’ TGTTCTCCTCAGAGCCTGAAC 3’  R:5’ TTTGTTTGACAGATCCTGAGCT 3’ | 65 |
| 104765 | F:5’ AGAAGACTGGACTGAAGATGATT 3’  R:5’ TAGTCTGCAGGATTAAATGTCC 3’ | 95 |
| β-actin | F:5' GTGGCCGAGGACTTTGATTG3'  R :5’ CCTGTAACAACGCATCTCATATT3’ | 73 |

**Table S3 . Primer sequences of circRNA_104761 Knockdown**

| *Name (siRNA)* | *Primer sequences* |
| --- | --- |
| siRNA-1 | F:5’ UCAGAGCCUGAACUGGCAUTT 3’  R:5’ AUGCCAGUUCAGGCUCUGATT 3’ |
| siRNA-2 | F:5’ CUCCUCAGAGCCUGAACUGTT 3’  R:5’ CAGUUCAGGCUCUGAGGAGTT 3’ |
| siRNA-3 | F:5' CCUGAACUGGCAUCAAAUATT 3'  R :5’ TAUUUGAUGCCAGUUCAGGTT 3’ |
